# Mothers' experiences of exclusive breastfeeding in a postdischarge home setting

**DOI:** 10.1111/mcn.13016

**Published:** 2020-04-21

**Authors:** Manya Van Ryneveld, Martha Mwangome, Jane Kahindi, Caroline Jones

**Affiliations:** ^1^ Centre for Tropical Medicine and Global health, Nuffield Department of Medicine Research Building University of Oxford Oxford UK; ^2^ Centre for Geographic Medicine (Coast) Kenya Medical Research Institute/Wellcome Trust Research Programme Kilifi Kenya; ^3^ School of Public Health University of the Western Cape Bellville South Africa

**Keywords:** breastfeeding, breastfeeding support, caregiving, infant feeding decisions, malnutrition, social factors

## Abstract

Re‐establishment and maintenance of exclusive breastfeeding (EBF) is recommended by the World Health Organization for the nutritional rehabilitation of malnourished infants under 6 months; however, there is no explicit guidance on how this should be achieved. The IBAMI study—a pilot study conducted in Kilifi, Kenya—implemented these recommendations using an intervention for hospitalized infants and their mothers that included ward‐based breastfeeding peer supporters. This paper explores how the challenges of maintaining EBF are recontextualized after infant hospitalization for malnutrition. Four weeks after discharge, semistructured interviews on experiences of trying to maintain EBF in a postdischarge home setting were conducted with a total of 20 mothers. Although most stated the aspiration of maintaining EBF for 6 months, a range of challenges were reported and not all had successfully maintained EBF post discharge. Reported challenges include the stress of household chores, food insecurity, technical difficulties and social stigma of expressing breast milk, pressure from neighbours and family members to introduce mixed feeding, and needing more community‐based awareness and support. Most of these challenges were specific to the home setting and were not easily surmountable, despite the breastfeeding practices mothers had learned in the ward. Indeed, in some cases, challenges were exacerbated by the overmedicalized nature of the breastfeeding practices taught in the ward. In order to aid the transition from ward to home, there may be a need to further translate ward‐based education and promotional messaging for EBF into a community setting, targeting other caregivers as well.

**Key Messages**

- Maintaining EBF in the context of infant hospitalization and subsequently in a postdischarge home setting is complex and contextual.
- Barriers and obstacles to EBF should be understood in relation to the broader social and cultural attributes of the mothers' environment, and interventions to promote EBF should be sensitive to these and should avoid becoming overmedicalized.
- Targeting EBF promotion to populations beyond pregnant women and mothers, including fathers and other caregivers, may facilitate the development of a more conducive environment for the continuation of EBF post discharge.

## 1 INTRODUCTION

In 2013, the World Health Organization (WHO) released updated guidelines addressing the management of severe acute malnutrition (SAM) in children and, for the first time, included a section on identifying and treating SAM in infants ‘under 6 months’ (U6M; WHO, 2013). These guidelines signalled a growing acknowledgement of the global prevalence of acute malnutrition in this age group and were the first attempt at bringing together what little evidence is available on its management. Central to the guidelines is the strong recommendation that the management of acute malnutrition in this age group should focus on establishing, or re‐establishing, exclusive breastfeeding (EBF; Kerac et al., 2012). Although these recommendations have been in circulation since 2013, in most low‐income settings, they are not consistently applied (Vygen, Roberfroid, Captier, & Kolsteren, 2013).

The rapid decline in EBF with increasing age is a common challenge and the difficulties of maintaining EBF throughout the first 6 months of life are increasingly acknowledged (Koosha, Hashemifesharaki, & Mousavinasab, 2008; Rollins et al., 2016). This recognition sits alongside (and sometimes in tension with) ongoing policy efforts to promote the practice of EBF as the fundamental component of infant nutrition. In community settings, the use of breastfeeding peer supporter (BFPS) has been shown to effectively increase the proportion of caregivers exclusively breastfeeding (Shakya et al., 2017), but the use of BFPS in an inpatient setting in low‐income countries has rarely been attempted. A recently published pilot study—the IBAMI study—on the implementation of the 2013 WHO guidelines amongst hospitalized malnourished infants U6M (Mwangome et al., 2019) introduced BFPS to facilitate the re‐establishment of EBF while the infants were inpatients in Kilifi County Hospital (KCH), Kenya. In addition to measuring nutritional rehabilitation outcomes, the IBAMI study included three substudies investigating (i) mothers' experiences of the process of re‐establishing EBF during admission; (ii) the perceptions of health professionals of working with BFPS in an inpatient setting; and (iii) mothers' experiences of trying to maintain EBF post discharge. The results from the first two substudies are reported elsewhere (Kahindi, Jones, Berkley, & Mwangome, 2019). The focus of this paper is the sub‐study exploring the experiences of mothers in trying to maintain EBF post discharge.

## 2 METHODS

### 2.1 Study site

The IBAMI study was implemented between September 2016 and January 2018 in the paediatric ward of KCH, located in coastal rural Kenya. Each year, approximately 150 infants aged below 6 months with low anthropometry are admitted to the KCH paediatric ward.

### 2.2 Selection of participants

A systematic random sample of mothers recruited with their infants into the IBAMI study (the first mother recruited each week) were invited to take part in the qualitative arm of the study. This involved participating in a first round of interviews at the point of discharge from KCH about their experiences of the IBAMI study (Kahindi et al., 2019) and a second, follow‐up round, 4 weeks after discharge. A total of 20 mothers were recruited to the qualitative arm, and the data from the second round of follow‐up interviews are the basis of this paper, which aims to explore their experiences of trying to maintain EBF at 4 weeks post discharge. The only exclusion criterion to participation was if the mother's infant had died during admission. There were no specific inclusion criteria, other than participation in the broader IBAMI study and exposure to its peer support intervention. However, in this analysis, data from two mothers who were not exclusively breastfeeding at discharge have been excluded.

### 2.3 In‐depth interviews

A structured interview guide with open questions was developed for use in the postdischarge interviews. The interviews were conducted in a quiet room on hospital grounds by an experienced local interviewer in a language identified to be comfortable for the participant: either Kiswahili, the national language of Kenya, or Mijikenda, the local language. With the consent of the participants, all but one of the interviews were digitally recorded. The interviews aimed to elicit information from the mothers about their experiences of trying to maintain EBF, whether they had been completely successful or not. Questions regarding daily feeding practices, major challenges, sources of support and recommendations for how mothers can be supported more were asked.

### 2.4 Data management and storage

Recorded interviews were downloaded onto an encrypted and password protected computer and subsequently deleted from the audio recorder. The digital files were stored in a folder that was only accessible to the study investigators. The digital recordings were transcribed and translated by the interviewer (J.K.) into English for the purposes of analysis. All transcripts were anonymized. Data were managed and organized using NVIVO version 10 and analysed by M.V. using thematic‐content analysis based on the coding charts. The coding and analysis was then corroborated with C.J. and M.M. Steps in the analysis process included familiarization with the transcripts, coding (using predefined codes relating to issues of interest to the authors such as ‘stigma’ or ‘education and community engagement’ and codes emerging from the transcripts), identification of themes, charting of themes and interpretation.

### 2.5 Ethical statement

Ethical clearance for the main (IBAMI) study was obtained from the Kenyan Medical Research Institute Ethical Review Committee in Nairobi (KEMRI/SERU/CGMR‐C/050/3285). Additionally, an information sheet was developed and shared at the beginning of each interview and gave the mothers an opportunity to ask further questions on the interview and seek clarification on other study related activities. Written informed consent was obtained from each participant before starting the interviews.

## 3 RESULTS

In total, 20 mothers were recruited at discharge, 18 of whom had successfully established EBF at discharge and two of whom had not. For the purpose of this analysis, which focuses on mothers' efforts to maintain EBF in a home setting, data from the two mothers who had not established EBF at discharge were excluded. The 18 mothers who had been exclusively breastfeeding at discharge reported that they had maintained either predominant breastfeeding or EBF, albeit with a range of challenges, during the 4 weeks post discharge. EBF is defined as feeding on breast milk only, whereas predominant breastfeeding is when breast milk is the predominant source of nourishment, with occasional supplementation from other sources such as water (WHO, 2015).

### 3.1 The ‘right’ feeding routine

When the infants were inpatients in KCH, expressing breast milk was a significant part of the daily breastfeeding routine, especially in the first 3 days after admission. Mothers were taught to express a predetermined volume of milk—calculated specifically for the child's nutrition plan—before attaching the child to the breast for a suckling feed and then finishing the feed with the expressed milk. This routine was conducted daily according to a strict time schedule, and BFPS would wake mothers up during the night to ensure they provided their babies with regular feeds. Once home, many of the mothers reported that this kind of routine became difficult to maintain. Several mothers said that they had adapted their breastfeeding schedule since discharge: breastfeeding either when they felt enough time had passed since the last feed or in response to their child's demands. This was often spoken about in terms of reading their child's body language to understand when their child was ‘satisfied’. For example, one mother said:

A parent knows that a child is satisfied in that, it's like when the child is crying you give the child the breast to breastfeed until the child releases the breast by herself and sleeps. Sometimes you see the child vomiting (burping) because maybe the child has breastfed a lot of milk, that's when you will know that the child is satisfied. (IDI2M037)

However, for a few of the mothers, their experience in the ward had resulted in a particularly ‘medicalized’ understanding of how to practice EBF. For these mothers, adhering to the strict feeding schedules and measuring predetermined volumes of milk at each feed remained important focal points of their home breastfeeding routines. This manifested in concerns with ‘being on target’ with breast milk production, having equipment to measure and store expressed milk and making sure that the child consumed the correct amount of milk at each feed—even if this meant forcing them to feed sometimes, or struggling with hand expression, syringes and measuring.

It's the child's cup, we came and measured the milk with a syringe, we took a syringe and poured the milk in the cup, and we knew where the 20mls mark is, when the cup is full its 30mls. But there is a mark when I pour the milk in the cup, I know this is 20mls. So I feed the child then I feed her 20mls again. The child was supposed to feed on 40mls (as per nutrition plan). (IDI2M001)

### 3.2 Challenges of expressed breast milk

The mothers had received support from the BFPS in the ward, which contributed to enabling them to express breast milk regularly during their time there. However, several mothers reported that they were no longer expressing as much once they returned home. Some had managed to strike a balance between breastfeeding and expressing, but almost all of them reported challenges with expressing at home, varying from pain, low production of milk, not having enough time, not having the right equipment and not being able to store the milk hygienically.

Many of the mothers reported that they were expected to perform a range of daily activities such as collecting firewood and water, or cooking and cleaning, which contributed to reducing the time available for breastfeeding. The expectation that they should express breast milk at every feed, rather than just suckle, exacerbated this sense of burden and many mothers either could not find time to express or were too tired to express, especially during the night.

It (expressing) is better during the day but at night, aah I'm usually tired. During the day the children have gone to school I have to do all the activities, because if the firewood is there then you wait for the cooking time, you know. And when I cook and finish then the children would have come. So when it reaches at night, I just sleep. (IDI2M043)

The data also suggest a difference in perceptions towards finding time to express breast milk as opposed to finding time to breastfeed directly. Breastfeeding directly was often reported as the more convenient option.

It's not the same (as in the ward) that's why I stopped. Because there you are seated and wait for the time you express and feed the child, but at home every kind of work is waiting for you. Children need to be cooked for, you need to wash clothes for the children and when you do not have water you need to go and fetch water, you go and fetch firewood. I said because the children (twins) are breastfeeding, let me just breastfeed them. (IDI2M021/022)

Negative perceptions towards expressed breast milk were reported from nine of the mothers, who had encountered varying degrees of negative responses from relatives and neighbours. One mother reported that her mother had commented that breast milk is a ‘dirty’ substance and refused to feed the baby with the expressed milk:

IDI2M010 She said: No just hold your own dirtiness. (laughter) Just hold your own dirtiness, I will not feed the baby.

M She will not hold the milk?

IDI2M010 No, she completely refused, she said you people are dirty, expressing and putting the milk in cups.

Another mother—described human milk as having a ‘fishy smell’ that might attract flies and lead to illness. A third mother said that many people talked about breast milk as ‘dirtiness’, but that because she had been educated (by the BFPS), she no longer saw it that way. However, she said she struggled to convince other people of this, and as a result, she could not get anyone to help her with feeding expressed milk to her child.

By contrast, two mothers reported that their partners were accepting of expressed breast milk:

M What of your husband, has he ever seen you expressing breastmilk?

IDI2M028 He has no problem even if he sees me.

IDI2M043 In the case that I'm away the father just takes the breastmilk and feeds the child when he is crying.

A few of the mothers spoke about expressed breast milk as part of a ‘treatment’ for their children's malnutrition leading to weight gain, and in one case, a mother mentioned that her husband saw her failure to express as a main cause of reduced weight of her baby since discharge:

Like the time the child was weighed and reduced weight, he (husband) used to tell me: ‘You were told to express breast milk for the child but you do not express the breast milk for the child. Look now the baby has reduced weight.’ (IDI2M017)

### 3.3 Insufficient breast milk and stress

A perception of insufficient milk production was a significant concern amongst the mothers. This was a persistent issue, having also been reported as a common breastfeeding challenge during the breastfeeding assessment taken at admission (Mwangome et al., 2019). Eight mothers described periods in the previous 4 weeks in which they were unable to produce sufficient milk. Another mother suggested that a perception of insufficient milk was a common reason why mothers in her community chose to discontinue breastfeeding early. Although many of the mothers reported concerns about insufficient milk, only two explicitly stated that they had introduced mixed feeding.

For the mothers, the issue of insufficient breast milk production was frequently linked to high levels of stress related to daily anxiety and ‘failing to get food’.

M Mmmh, so you fail to get food sometimes?

IDI2M030 Eeeh (yes)

M has failing to get food been disrupting with the breast milk production?

IDI2M030 Its disrupts because the milk production will not be what you would want to produce

During their time in the hospital, the mothers had been exposed to educational messages about the need to manage stress and have a good diet in order to enhance milk production. In the hospital, the women had been provided with food, and emphasis had been placed on the importance of relaxing while breastfeeding in order to stimulate milk (Mwangome et al., 2019). At the postdischarge interviews, many of the women were aware of how stress hindered their ability to produce sufficient milk and spoke about relieving themselves of stress, often through prayer, or ‘taking heart’.

M What strategies are you using to address your stress problems?

IDI2M001 I just take heart, I say I will come over this, and when I take heart I see I have relaxed and then the milk gets back/I start producing milk.

### 3.4 Community pressure to introduce mixed feeding

Perceptions around the insufficiency of breast milk were compounded by external pressure from neighbours and relatives to introduce other foods, which in turn contrasted starkly with the lessons learned in the ward where the introduction of alternative foods in lieu of EBF was frowned upon. Pressure to introduce other foods came from different quarters and with different justifications but was generally related to a perception of a dissatisfied child. Many mothers reported being told that their child would stop crying if they were fed porridge.

IDI2M040/41 They talk a lot

M Who are these people talking?

IDI2M040/41 Neighbours, they say children are taking the whole night crying, why do not you prepare porridge for them, when you give the children porridge they will stop crying.

Other mothers were told by neighbours that feeding breast milk alone was not enough, with some mothers even reporting that they had been accused of leaving their child to go hungry or thirsty:

Though they hear about that (EBF) but they will say ‘eeeeh, you are being lied to!’ They say … how will you leave your child to stay that way, you do not even give the child water, you are starving the child with thirst. (IDI2M006)

Many of the mothers reported that awareness of EBF in the community was low, and little was communicated about it through antenatal care services. Resisting the pressure from relatives and neighbours was said to be particularly difficult when it came with accusatory sentiments that questioned a mother's parenting abilities.

### 3.5 Opportunities for postdischarge care and follow‐up

The advocacy amongst neighbours and relatives for the early introduction of supplementary liquids and foods was often attributed to a lack of knowledge of EBF in the wider community and missed opportunities to disseminate this knowledge through antenatal care services. Although breastfeeding and EBF counselling is supposed to be offered to mothers during antenatal care, the general opinion reported in the data was that this service was not always given, and if it was, the practice was not emphasized or explained well to mothers.

They (nurses at the antenatal clinics) are talking, but not deeply. They say do not give the children water, do not do this but they do not stress on it … IDI2M023

One respondent noted that there are mothers who have no opportunity to hear of the importance of EBF as they do not attend antenatal care sessions or give birth in hospital.

That is after you expand the study, you should visit people at home, ask the mothers where is your home, they tell you where they come from. Then you go to those homes and then you explain people on what you are doing. Explain yourself to them, that's when the mother will understand because there are other mothers who do not even go to the clinic. (IDI2M023)

There was a recurring request from the mothers for continued follow‐up at home over the full 6 months of recommended EBF. Continued follow‐up was described not only as a way of encouraging mothers to keep up their EBF practices but also as a way of making the practice more visible in the community. The suggestion was made to use follow‐up sessions as community engagement opportunities, or to hold meetings in mothers' houses, where knowledge could be disseminated to other mothers in the community.

If you have a chance, you should come and see the child at home, if you do not have time then you can call and ask the child's progress. For me I think it's good if there will be some follow‐ups so as we would be asked the progress of the child, is the child getting enough breastmilk or what is happening? So the parent should be visited after every month. (IDI2M045)

One mother emphasized the need for holding these kinds of meetings in a public space in order to involve other caregivers, especially fathers, who may normally be uninvolved in such activities:

I think you can come and hold some meetings, for example if we hear there is a meeting in Kibaoni Primary and we are educating mothers. When the fathers would be passing near the meeting then they would go and inform their wives incase their wives would not be able to attend the meeting. (IDI2M001)

### 3.6 Sources of support

Towards the end of the interviews, mothers were prompted to reflect on the sources of support from families and communities they received on returning home and continuing with EBF. These questions yielded rich data on the role of relational networks in shaping and motivating the mothers' breastfeeding practices. Immediate family support, primarily from their mothers, husbands and older children, was mentioned by all the mothers as their main source of support and was acknowledged as an important factor in their motivation to continue with EBF.

IDI2M001 The one supporting me in feeding the child is my mother. If I will not feed the child, my mother is the one who is feeding the child. Sometimes the child's father.

M How is he (child's father) supporting you?

IDI2M001 Money, when I tell him to assist me with money he gives me.

A few mothers reported that their husbands would assist in holding the child while they completed other chores or by doing chores for them:

He supports me in providing food for me and when the children (twins) are crying, he assists in calming the children. He is also fetching water for me from the tap, other times when the children (older children) are not around he also cooks food. (IDI2M040/41)

There was a clear gender dynamic in the kinds of support offered by family members, with husbands reported to provide support in the form of food, clothes and money, whereas mothers and older children often provided support in the form of childcare and taking over chores.

## 4 DISCUSSION

At 4 weeks post discharge, all 18 of the mothers who had been practicing EBF at discharge reported maintaining predominant breastfeeding or EBF, but they also talked of facing a wide range of challenges in maintaining EBF in the home setting. These challenges can be broadly characterized as concerns about insufficient milk, ability to accurately reproduce what had been taught in hospital, coping with the technical difficulties and social stigma of expressing breast milk and finding support amongst family and community members for EBF. Several of these issues have been identified in previous studies on the challenges to achieving and maintaining EBF in ‘well’ infants (Kimani‐Murage et al., 2015; Nduna, Marais, & van Wyk, 2015; Talbert, Tsofa, Mumbo, Berkley, & Mwangome, 2018), but this study identified additional concerns that relate to the hospitalization of the infant and the context within which efforts to re‐establish EBF took place. Understanding the intricate and variable processes of negotiation that many of the mothers undertook in order to ‘achieve’ EBF at home suggests that continuity of EBF was strongly desired by many of the mothers but hindered by the change of context.

### 4.1 The risks of over‐medicalization

A key, and perhaps overlooked, aspect of continuity of care post discharge is the ability of the patient (or, in this case, carer) to recreate at home what they have been taught in the controlled and medicalized environment of a hospital. This is explored to some degree in the literature on discharge planning, as well as in literature critiquing the responsibility placed on home caregivers who are often untrained family members (Bull, 1992; Shepperd et al., 2013). However, there is little on the experiences of learning breastfeeding techniques in the context of infant hospitalization and transitioning this knowledge into a home setting. The results in this study suggest that the mothers' experiences of EBF during the ward‐based intervention played a role in informing their expectations of how EBF should be practiced at home and that, in some cases, this created a dissonance leading to further challenges.

The data suggest that, for many of the mothers, breastfeeding and breast milk—two inherently ‘natural’ things—shifted materially and conceptually into the realm of the biomedical, as a result of their time in the ward. Attention became focused on breast milk as a medicinal substance needing to be measured, expressed and kept sterile, rather than the product of an intimate process between a mother and child (Lee, 2018). Tacit knowledge such as ‘knowing when your child is satisfied’ was not explicitly valued in this conceptualization of breastfeeding, even if some mothers continued to use it in their feeding practices.

Given the graveness of experiencing a hospital admission for a severely ill child and the emphasis placed on EBF as a fundamental part of the treatment plan, it is understandable that the mothers' perceptions of breastfeeding and breastfeeding techniques appear to have been substantially impacted. This may also have been compounded by the fact that many of the mothers reported receiving very little information about EBF before the IBAMI study (e.g., during antenatal care visits or immediately after delivery) and most had not practiced EBF with their older children.

The association between their child's illness experience and the practice of EBF as a ‘treatment’ can be regarded as an unintended consequence of the way in which the IBAMI study was conducted. This has important implications for the way in which knowledge around EBF is produced and shared beyond the ward, particularly in light of a contextual setting in which EBF up to 6 months is a relatively uncommon practice or, in the case of breast milk expression, associated with alternative (and sometimes negative) lay understandings (Talbert et al., 2018). Breastfeeding shifted from a low‐tech, ‘natural’ process to a ‘technologized intervention’ through the experience of relearning EBF in the ward, where emphasis is placed on scheduling, measurement and equipment (Slusser & Frantz, 2001; Torres, 2014). Responsibilities towards hygiene, routine and comfort, often perceived to belong solely to the mother, could inadvertently undermine the ‘low‐tech’ nature of EBF and exacerbate perceptions of stress, anxiety and maternal inadequacy (Chary, Dasgupta, Messmer, & Rohloff, 2011). Lastly, the emphasis placed on measuring prescribed volumes of breast milk in the ward added pressure to mothers who then saw themselves as having insufficient supply if they failed to produce the ‘correct’ volume of milk once home.

Given that one of EBF's major strengths as a public health intervention lies in its simplicity and accessibility, the implications of overmedicalizing its practice through clinical guidelines and antenatal care messaging should be explored further. This study highlights the importance of understanding the translation and uptake of biomedical definitions and standards of EBF, as stipulated in clinical nutritional guidelines and taught in ward and antenatal care settings, once mothers return home. Acknowledging how this interacts with other social and structural challenges that mothers face in practicing EBF at home—from negotiating the daily stresses of household chores, to grappling with food and income insecurity—could prove useful in mitigating the risk of EBF sliding off the routine once mothers return home.

### 4.2 Understanding EBF in the home environment

The results of this study suggest that when mothers moved out of the artificial ward environment, the interaction of their lived realities with the practices they had learned in the ward became a crucial determinant of whether they were able to maintain EBF. Understanding and unpacking these interactions is fundamental to improving support for EBF. A socioecological framework may be useful for conceptualizing the underlying causes of barriers and obstacles to EBF, integrating them with a much wider set of environmental, socio‐economic and cultural circumstances that operate at the individual, group and societal level (Bueno‐Gutierrez & Chantry, 2015; Hector, King, Webb, & Heywood, 2005; Rollins et al., 2016).

For example, a perception of insufficient milk is one of the commonest reasons women give for stopping breastfeeding, even though evidence suggests that physiological obstructions to breast milk production are present in less than 5% of women (Vygen et al., 2013). This mirrors studies conducted in other LMIC settings, where insufficient breast milk is reported as one of the main obstacles to EBF (Khatun et al., 2018; Nduna et al., 2015). Although an insufficiency of milk was widely reported by the mothers in this study, understanding the circumstances behind this may be more valuable than simply interpreting this insufficiency at face value. As Hector et al. (2005: 56) point out, ‘the explanation of “insufficient milk” can mask a range of underlying factors that undermine breastfeeding’. As the results of this study show, the perception of insufficient breast milk can be seen as a proxy for a wide range of other socioecological issues—such as stress related to food and/or income insecurity, or the pain, physical discomfort and stigma related to breastfeeding and/or expressed breast milk.

The interaction with the IBAMI study processes and the BFPS provided the mothers with exposure to guidance and advice on EBF that may not have been easily accessed normally. Although much of the advice that the mothers received in the ward was technical, there was also an important element of feeling supported by a community of peers (Kahindi et al., 2019). Replicating these sources of support outside the ward environment may be challenging. Although breastfeeding is largely an accepted and normal part of infant feeding practices in Kenya, the idea of ‘exclusive’ breastfeeding for the first 6 months is often perceived as extreme and garners far less acceptance (Matsuyama, Karama, Tanaka, & Kaneko, 2013; Talbert et al., 2016). This is compounded by the normalization of introducing water, herbal teas or watered‐down porridge to ‘quench thirst’—a fairly common practice present in this study's setting and in settings across Africa and South America (Giugliani, do Espírito Santo, de Oliviera, & Aerts, 2008; Nduna et al., 2015). Additionally, as the data shows, expressing breast milk was not easily accepted outside of the ward—the idea that expressing and storing breast milk could save mothers time and allow them to return to work while maintaining EBF, as has been cited in some literature, particularly from high‐income settings (Clemons & Amir, 2010), did not seem to have much traction with the mothers.

Many studies looking at barriers to EBF cite the influence of community elders—usually grandmothers and older mothers—as negatively contributing to EBF, particularly amongst first‐time mothers (Khakoni‐Walingo & Amanya Mutuli, 2014; Talbert et al., 2016). In cases where fathers are relatively absent from caregiving practices, maternal elders are often the only source of support and knowledge for breastfeeding mothers. They may also play caregiving roles once mothers return to work and therefore yield significant influence over feeding practices. This presents a difficult conundrum for EBF promotion. Shifting the dynamics of family, community and social support that are available to mothers practicing EBF may be largely contingent upon enabling social and cultural acceptance of EBF—through community engagement and meetings, as many of the mothers in the study suggested. However, it is arguable that these shifts may also only take place alongside changes in other social and economic factors that are drivers of behavioural and attitudinal change (Wallace & Adongo, 2018). By paying attention to possible changes in attributes such as gender dynamics, maternal autonomy, food security and the familial division of labour in caregiving, future interventions may be better positioned to leverage support for EBF amongst family, community members and broader society. In other words, it is necessary to highlight the social and structural barriers to EBF, in order to determine effective ways of expanding support for exclusively breastfeeding mothers and spreading the share of responsibility for achieving EBF to fathers, other caregivers and the broader community (Renfrew, Mccormick, Wade, & Quinn, 2014).

## 5 CONCLUSION

Despite having a desire to do so, maintaining EBF post discharge can be challenging for mothers whose babies have been hospitalized with malnutrition. Many of the day‐to‐day obstacles women come up against at home are not easily surmountable, despite the practices they learn in the ward. The ward environment itself may create its own problems with the risk of overmedicalizing the techniques, hindering continuity of EBF in the home. Targeting EBF promotion efforts at populations beyond pregnant women and mothers, including fathers and other caregivers, particularly elder women who play a fundamental role in passing on knowledge about child care and nutrition to younger mothers, may facilitate the development of a more conducive environment for the continuation of EBF post discharge. It is clear from the data that family and community members play crucial roles as sources of support and influence in the mothers' ability to practice EBF. This should be leveraged in future EBF promotion efforts, which should take place in community settings rather than in hospitals alone.

## CONFLICTS OF INTEREST

The authors declare that they have no conflicts of interest.

## CONTRIBUTIONS

MM and CJ designed the study and data collection tools. MM and JK implemented the study and collected and managed the data. MVR, MM, and CJ analysed the data and drafted the initial manuscript. All authors have revised and approved the final manuscript.
